# Oligoimide-Mediated Graphene Oxide-Epoxy Nanocomposites with Enhanced Thermal Conductivity and Mechanical Properties

**DOI:** 10.3390/mi13091379

**Published:** 2022-08-24

**Authors:** Muhammad Inshad Khan, Toheed Akhter, Humaira Masood Siddiqi, Young Jun Lee, Hyeonjung Park, Muhmood ul Hassan, Chan Ho Park

**Affiliations:** 1 Department of Chemistry, Quaid-i-Azam University, Islamabad 45320, Pakistan; 2 Department of Chemical and Biomolecular Engineering, Korea Advanced Institute of Science and Technology (KAIST), Daejeon 34141, Korea; 3 Department of Chemistry, School of Science, University of Management and Technology, Lahore 54770, Pakistan; 4 NQE, Korea Advanced Institute of Science and Technology (KAIST), Daejeon 34141, Korea; 5 Department of Chemical and Biological Engineering, Gachon Univeristy, Seongnam 13120, Korea

**Keywords:** epoxy, nanocomposites, thermal conductivity, oligoimide linker, modified graphene

## Abstract

The current study reports the preparation of thermally conductive polymeric nanocomposites. For this purpose, two epoxy-based nanocomposites were prepared by dispersing a different type of functionalized graphene oxide (GO) nanofiller in each series. Both these GO nanofillers were functionalized by covalently bonding oligoimide chains on their surfaces. In one series, these oligoimide chains were prepared by reaction of 3,3′,4,4′-benzophenonetetracarboxylic dianhydride (BTDA) with a diamine 4,4′-methylenedianiline (MDA). While in the other case, BTDA was reacted with N,N′-[((propane-2,2-diylbis(4,1-phenylene))bis(oxy))bis(4,1-phenylene)]bis(4-aminobenzamide) (BDM) to mount oligoimide chains on the surface of GO. Both types of oligoimide chains have amino groups as chain-end functional groups. These modified GO nanofillers were added to the epoxy matrices separately to prepare their respective nanocomposites (MDA-B-GO-epoxy nanocomposites and BDM-B-GO-epoxy nanocomposites). The chain-end amino groups of oligoimide chains reacted with the epoxy ring developing a covalent bonding between oligoimide chains of GO and the epoxy matrix. Moreover, these oligoimide chains prevented the agglomeration of GO by acting as spacer groups leading to the uniform dispersion of GO in the epoxy matrix. Various analytical techniques were used to examine the attachment of oligoimide chains to the GO surface, and to examine the morphology, curing potential, mechanical strength, thermal stability, and thermal conductivity of the prepared nanocomposites. We demonstrated that the thermal conductivity of MDA-B-GO-epoxy nanocomposites increased by 52% and an increase of 56% was observed in BDM-B-GO-epoxy nanocomposites. Similarly, a significant improvement was observed in the mechanical strength and thermal stability of both types of nanocomposites.

## 1. Introduction

The formation of polymer nanocomposites by incorporating nanofillers in polymer matrices has been studied for decades due to their extraordinary enhancement in chemical and mechanical properties [1,2]. In particular, epoxy-based nanocomposites have various industrial applications achieving high chemical resistance, excellent adhesive characteristics, good corrosion resistance, and little shrinkage in the curing process [3]. However, epoxy resins inherently possess brittleness and low thermal conductivity, which result in crack propagation and poor heat dissipation in their final nanocomposites. These drawbacks lower the service temperature and device stability in applications, including electronic equipment [4,5].

Such problems of epoxy resin are mitigated by the incorporation of nanofillers that can decrease the brittleness and improve the heat-dissipating ability [6]. Graphene oxide (GO) is the most widely used two-dimensional nanomaterial, which has shown good potential for improving electrical, mechanical, and thermal properties of epoxy-based nanocomposites [7]. The improvement in the properties of nanocomposites is attributed to the high surface area, high inherent mobility, Young’s modulus, large mechanical stiffness, and good thermal conductivity of GO [8,9]. 

Maximum property enhancement in nanocomposites by the addition of GO filler can be achieved by ensuring the following: (i) exfoliation of graphene sheets in the polymer matrix so that it can transfer its intrinsic characteristics to composite materials and (ii) strong interfacial interaction with the matrix for effective load distribution in the nanocomposites [10]. These two points can be fulfilled by functionalizing GO with chemical moieties that can interact strongly with a matrix. For example, functionalization of the GO surface with amines can be effective because amino groups are efficient epoxy curing agents. 

Chatterjee et al. modified graphene nanoplatelets with dodecyl amine and reported that the fracture toughness of the epoxy nanocomposite was increased 66% by incorporating 0.1 wt % of modified graphene nanoplatelets [11]. However, they did not show the effect of this modification on thermal conductivity of nanocomposites. Ribeiro et al. reported an increase of 72% in Young’s modulus and an increase of 143% in hardness upon the incorporation of 0.5 wt % of amino-functionalized GO. They also reported a significant increase in thermal conductivity, but did not study the degradation behavior of nanocomposites. Similarly, Yu et al. modified GO with diamine functionality and reported a significant increase in the cross-linked density, glass transition temperature (*T*_g_), and mechanical properties of prepared nanocomposites. However, they did not study degradation behavior of these nanocomposites [12]. Likewise, Liu et al. grafted the epoxy monomers (DER332) and the curing agent diaminodiphenyl methane onto the GO surface and prepared GO-epoxy nanocomposites using different loading of this modified GO [13]. They reported 62% increase in the storage modulus and 26 °C enhancement in the *T*_g_ upon 0.2 wt % addition of the modified GO to the epoxy matrix. However, it was observed that tensile strength, *T*_g_, and storage modulus decreased when GO was loaded beyond this concentration. Thangaraj et al. functionalized GO with hexamethylenediamine and added this modified GO to the epoxy matrix in various concentrations i.e., 0.2–0.6 wt % [14]. They observed that optimum concentration of this modified GO was 0.4 wt % where both modulus and T_g_ of epoxy increased from 2385 MPa to 3810 MPa and 109 °C to 122.27 °C, respectively. However, after this concentration both modulus and *T*_g_ decreased.

In this work, two types of surface-functionalized GO were prepared by chemically attaching oligoimide chains to their surfaces. In the one type, which is called MDA-B-GO, oligoimide linkers were affixed to the GO surface by a reaction of 3,3′,4,4′-benzophenonetetracarboxylic dianhydride (BTDA) with a diamine 4,4′-methylenedianiline (MDA). Whereas, in the other type, which was coded as BDM-B-GO, oligoimide chains were attached to its surface by reacting BTDA with *N,N*′-[((propane-2,2-diylbis(4,1-phenylene))bis(oxy))bis(4,1-phenylene)]bis(4 aminobenzamide) (BDM). The surface modification of GO in both cases was analyzed by FTIR and XPS analysis. The oligoimide moieties, on the surface of GO, contained amine end-functional groups. We hypothesized that these amino groups could cure the epoxy rings, and, thereby, could develop covalent bonding between the GO nanofiller and epoxy matrix, which was confirmed by evaluating the curing potentials of MDA-B-GO and BDM-B-GO by DSC analysis. Then, both these oligoimide modified GOs were dispersed separately in the epoxy matrix to prepare two types of epoxy nanocomposite series, MDA-B-GO-epoxy nanocomposites and BDM-B-GO-epoxy nanocomposites. As a reference, a neat epoxy system was also prepared by curing the same epoxy resin with a commercial harder Aradur-22962. We observed that both types of modified GO were able to disperse well, develop chemical bonding and enhanced the curing process of epoxy resin. Consequently, both nanocomposite systems performed with high thermal conductivity, thermal stability, and mechanical strength.

## 2. Experimental

### 2.1. Materials and Measurements

All the chemicals and reagents were of analytical grade and were used as received, unless otherwise mentioned. All chemicals were purchased from Sigma Aldrich (St. Louis, MO, USA), Fluka (Darmstadt, Hesse, Germany), and Merck (Kenilworth, NY, USA). Diamine BDM was synthesized as reported in our previous article [15]. (3-Aminopropyl) trimethoxysilane (APTMS, 97%), acetone (99.5%), 4,4′-methylenedianiline, MDA (97%) and 3,3′,4,4′-benzophenonetetracarboxylic dianhydride (BTDA, 98%) obtained from Sigma Aldrich, South Korea and were used as received. N-Methylpyrrolidone (NMP, 99%) was provided by Merck, South Korea. Huntsman epoxy resin Araldite LY 564 having 0.497 epoxy value equivalence/100g and hardener Aradur-22962 (cycloaliphatic polyamine) were used. The solvents used in the presented work were purified and dried using standard procedures [16]. 

### 2.2. Instrumentation

Fourier transform infrared (FTIR) spectra were recorded on an a-Alpha-P model (Bruker, Zurich, Switzerland) using the attenuated total reflection (ATR) method. Thermogravimetric analysis (TGA) was carried out using a TGA N-1000 Thermo gravimetric Analyzer (TA instruments, New Castle, DE, USA). Differential scanning calorimetric (DSC) analysis was carried out on the DSC 25 TA instruments by keeping a scanning rate of 10 °C min^-1^ under an inert atmosphere. X-Ray photoelectron spectroscopy (XPS) spectra were recorded on XPS machine model k-Alpha (Thermo VG Scientific, Waltham, Massachusetts, USA). The surface morphologies of the prepared nanocomposites were studied by scanning electron microscopy (SEM) (Magellan 400, FEI Company, Hillsboro, OR, USA) with an accelerating voltage of 5kV. Thermal conductivity was measured by the hot disk method using TPS 2500 S. Nanoindentation tests were carried out using Nano indenter XP (SURFACE systems technology GmbH & Co, Hückelhoven, Germany) (iNano Nano indenter with Berkovich diamond tip). X-Ray analysis was carried out on the RIGAKU D/MAX-2500 X-ray diffractometer.

### 2.3. Surface Modification of Graphene Oxide

**Scheme 1:**
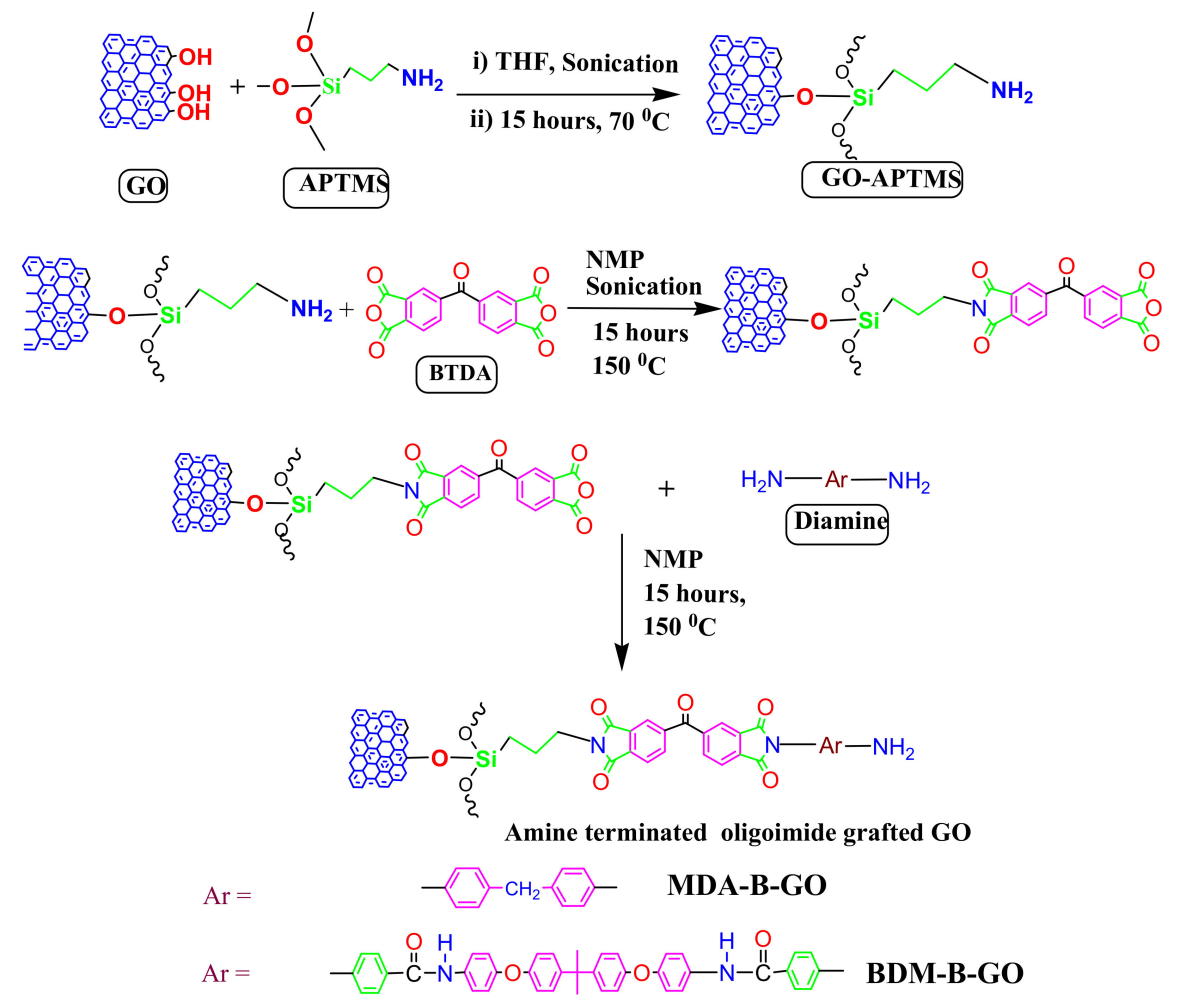
Surface modification of graphene oxide (GO).

The surface modification of synthesized graphene oxide (GO) was carried out using BDM, MDA, and BTDA following a reported procedure [15], as given in Scheme 1. 

For this purpose, GO was dispersed in THF (0.1 mg/mL) at room temperature by sonication for 1 h. To this dispersion, APTMS was introduced (GO:APTMS, 1:10) and the mixture was heated at 70 °C for 15 h. Afterwards, the resulting mixture was filtered and washed numerous times with THF to completely eliminate unreacted APTMS. The APTMS-functionalized GO was dried for 48 h in the vacuum oven. This dried APTMS-modified GO was then dispersed in anhydrous NMP (0.1 mg/mL) by sonication for 1 h to get homogenous dispersion. To this well-dispersed mixture, 3,3′,4,4′-benzophenonetetracarboxylic dianhydride (BTDA) was added (APTMS-modified GO:dianhydride, 1:10) and refluxed again for 15 h. This mixture was allowed to cool to room temperature and anhydride terminated GO was obtained by filtration. After washing the residue several times with NMP and drying under vacuum for 24 h, it was again dispersed in anhydrous NMP. Then, the diamines (GO: diamine, 1:10) were added separately and the mixture was refluxed for 15 h to get functionalized GO with imide groups and amine end groups. The oligoimide synthesized using MDA and dianhydride BTDA was coded as MDA-B-GO, while the oligoimide obtained by reaction of diamine BDM was given the code BDM-B-GO.

### 2.4. Preparation of MDA-B-GO and BDM-B-GO-Epoxy Nanocomposites

MDA-B-GO and BDM-B-GO-epoxy nanocomposites were prepared separately by the solution mixing method. As an example, the preparation of MDA-B-GO-epoxy nanocomposites is discussed here. The various amounts (1, 3, and 5 wt %) of MDA-B-GO were dispersed (in three separate caped containers) in acetone by sonication for 2 h. A calculated amount of the commercial epoxy LY-564 was added to each suspension followed by sonication for another two hours. Then, the solvent was evaporated by gentle heating and a calculated amount of hardener aradur-22962 was added to each mixture. All the mixtures were subjected to shear mixing for 15 min to achieve homogeneous dispersion of hardener. After proper mixing of hardener, the entrapped air bubbles were removed from mixtures by placing them under vacuum for an adequate time. These mixtures were then transferred to the molds and were cured by heating them for one hour at 40 °C, 80 °C, 100 °C, and 120 °C, and for two hours at 150 °C. Along with these GO-epoxy nanocomposites, neat epoxy network (without the addition of MDA-B-GO and BDM-B-GO) was also prepared using the aforementioned procedure for the properties’ evaluation. Furthermore, a similar procedure was adopted to prepare BDM-B-GO-epoxy nanocomposites. The recipe and codes of prepared nanocomposites are given in Table 1.

## 3. Results and Discussion

### 3.1. Surface Modification of GO

To modify the surface of GO with the diamines (as shown in Scheme 1), first APTMS was attached to it by the condensation reaction between hydroxyl groups of GO and trimethoxysilyl groups of APTMS, leading to the preparation of GO-APTMS with amine end-functional groups. BTDA was then reacted with GO-APTMS, which affixed imide linkages and converted amine end-functional groups to anhydride end-functional groups on GO. Afterwards, diamine (MDA, BDM) was reacted with this intermediate which resulted in the attachment of oligoimide moieties on the surface of GO. Two differently modified GOs, MDA-B-GO and BDM-B-GO, were prepared. This surface modification of GO was confirmed by the FTIR and XPS analyses. 

**Figure 1:**
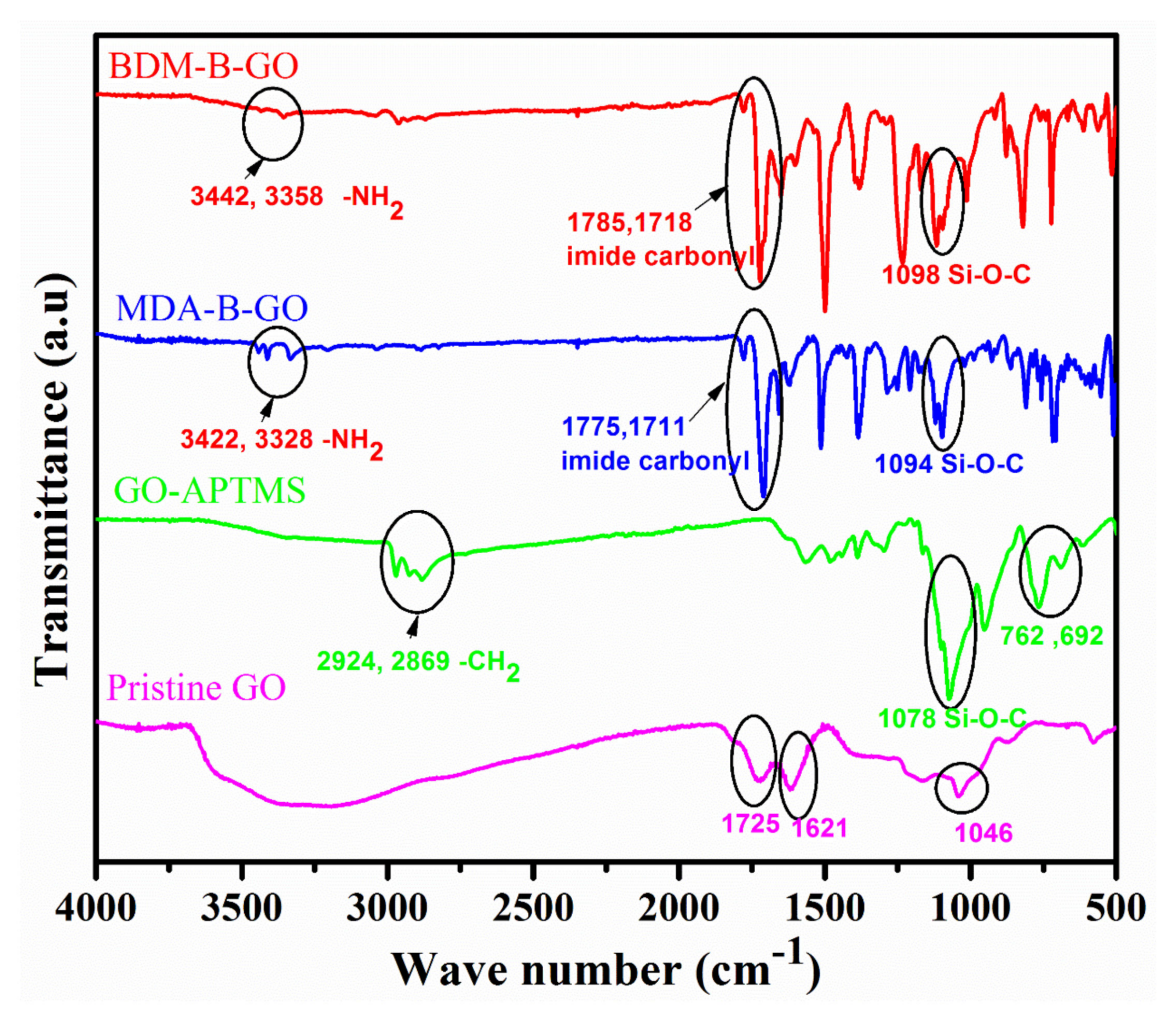
FTIR spectra of GO, GO-APTMS, MDA-B-GO and BDM-B-GO.

The FTIR spectra of various stages of GO modification are represented in Figure 1. In the FTIR spectrum of pristine GO, characteristic absorption bands of C=O and C-O stretching vibrations of –COOH were observed at 1725 cm^−1^ and 1046 cm^−1^, respectively. The attachment of APTM on the GO surface, at the first step of the modification, was authenticated by the presence of C-H stretching vibration peaks of the methylene group, N-H bending peaks, and Si-O-C stretching vibration peaks in the FTIR spectrum of GO-APTMS. The asymmetric and symmetric stretching vibration bands of the methylene groups were observed at 2924 and 2869 cm^−1^, respectively, while N-H bending peaks were present at 1563cm^−1^ and 762 cm^−1^_._ The peaks at 1078 and 692 cm^−1^ were attributed to Si-O-C stretching. More importantly, the presence of oligoimide groups on the surface of GO was confirmed by asymmetric and symmetric stretching vibration peaks of imide carbonyl groups. These peaks were observed at 1775 and 1711 cm^−1^ in the case of MDA-B-GO, and at 1785 and 1718 cm^−1^ for BDM-B-GO. Further confirmation came from the peaks at 3422 and 3328 cm^−1^ (in MDA-B-GO), which were attributed to amino end-functional groups of oligoimide moieties. These peaks were present at 3442 and 3358 cm^−1^ in the FTIR spectrum of BDM-B-GO. 

**Figure 2:**
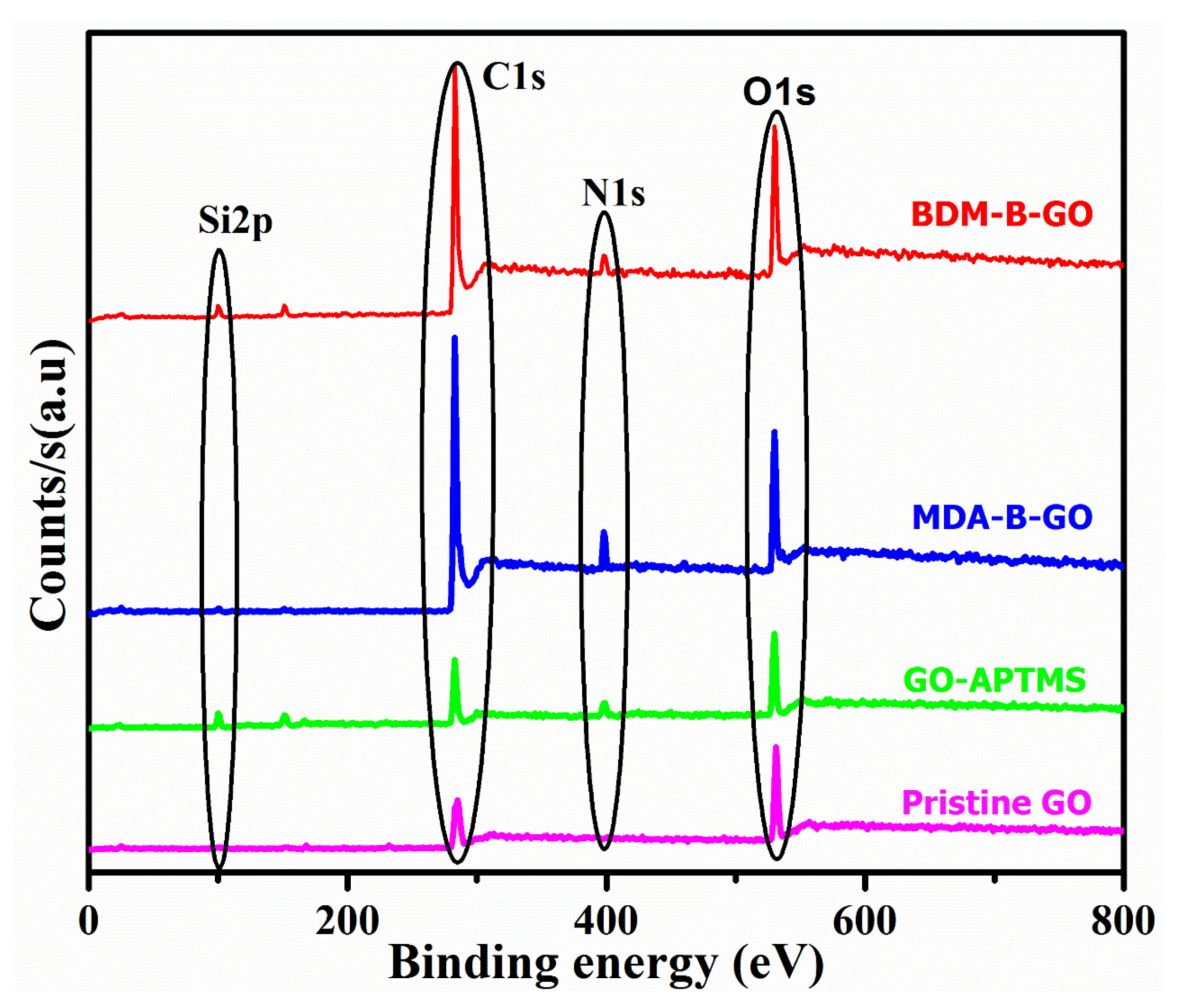
XPS spectra of GO, GO-APTMS, MDA-B-GO and BDM-B-GO.

The covalent grafting of oligoimides to the surface of GO was also confirmed by XPS analysis. The XPS spectra are shown in Figure 2. It can be seen that the XPS spectrum of pristine GO did not show the peaks for Si2p and N1s. These peaks are, however, clearly present in the XPS spectra of GO-APTMS, MDA-B-GO, and BDM-B-GO, which corroborates the bonding of APTMS and amine terminated oligoimides on the surface of GO. Furthermore, the peak intensities of N1s and O1s increased in the case of MDA-B-GO and BDM-B-GO, as compared to GO-APTMS and pristine GO.

The in-depth results of the XPS analysis are also summarized in Table 2. As is obvious from Table 2 the carbon to oxygen and carbon to silicon ratios in both types of modified GOs are much higher than GO-APTMS and pristine GO, which endorses the successful modification of GO. Higher values of these ratios for MDA-B-GO than BDM-B-GO can be attributed to the high solubility and processability of MDA diamine, as compared to BDM diamine.

### 3.2. Curing Potential of MDA-B-GO and BDM-B-GO

**Figure 3:**
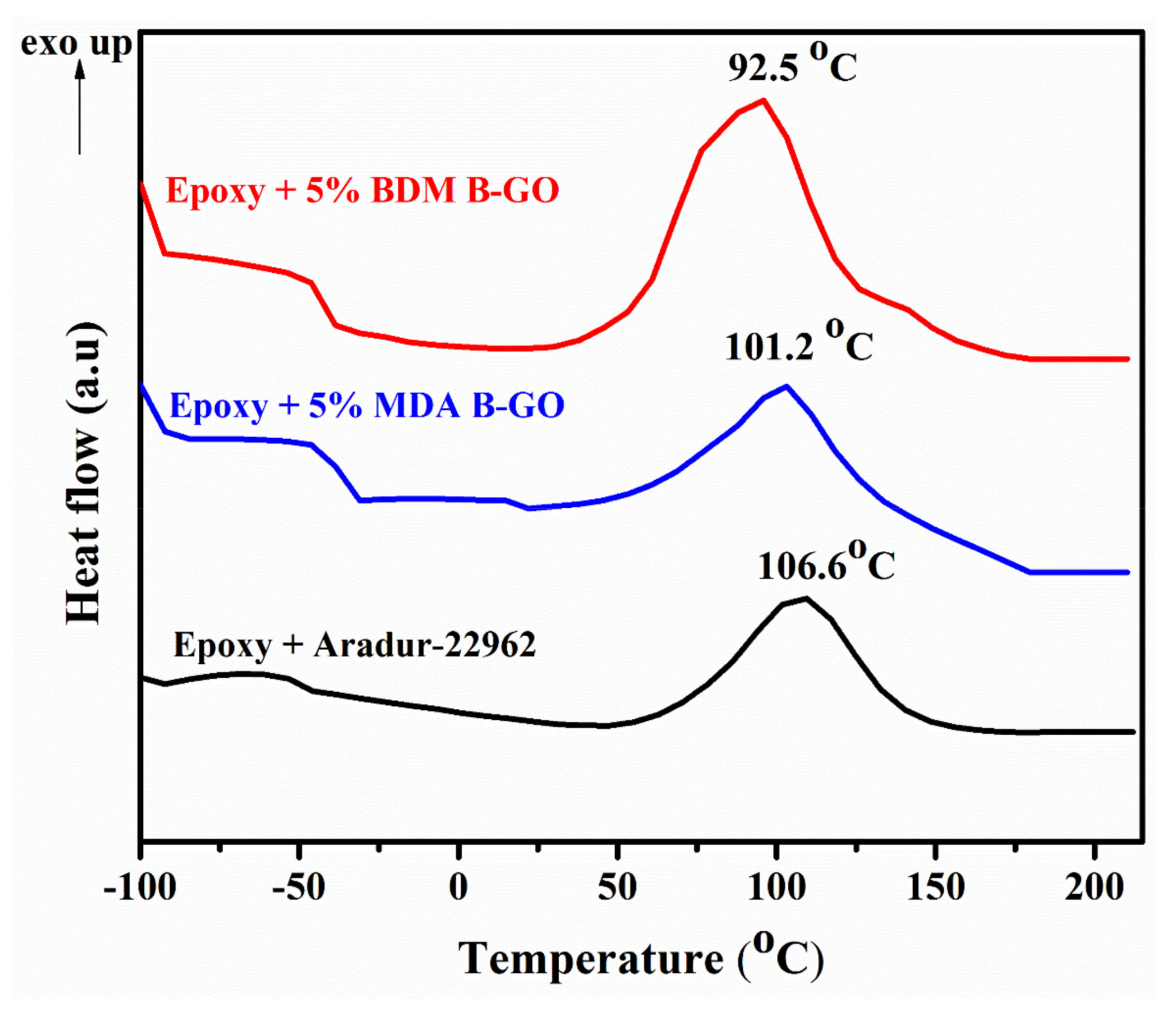
DSC thermograms of epoxy with oligoimides MDA-B-GO and BDM-B-GO with heating rate of 10 °C /min.

As confirmed by FTIR and XPS analyses, both MDA-B-GO and BDM-B-GO contained amine end-functional groups which can cure the epoxy resin. So, the curing aptitude of both modified GOs was studied using DSC, and their thermograms are presented in Figure 3.

In order to assess the curing ability of MDA-B-GO and BDM-B-GO, their curing behavior was compared with data obtained from the curing of epoxy resin with a commercial hardener aradur-22962. As exhibited in Figure 3, a single exothermic peak with different maximum peak temperature (*T*_max_) was presented in all the DSC thermograms. The *T*_max_ values for the epoxy-GO nanocomposites having 5 wt % MDA-B-GO and 5 wt % BDM-B-GO were found to be 101.2 °C and 92.5 °C, respectively, while for the neat epoxy it was 106.6 °C. Moreover, ∆H values reduced to 76.6 and 45.7% when MDA-B-GO and BDM-B-GO were used for curing, respectively, in comparison to curing with Aradur-22962. So, a single exothermic peak (*T*_max_) at low temperature for both nanocomposites and reduced ∆H values, compared to those of neat epoxy, confirmed the curing aptitude of both MDA-B-GO and BDM-B-GO, due to the presence of the terminal −NH_2_ groups. The detailed curing data of both nanocomposites and neat epoxy are given in Table 3.

## 4. Characterization of Epoxy Nanocomposites

### 4.1. FTIR of Neat Epoxy and Nanocomposites 

**Figure 4:**
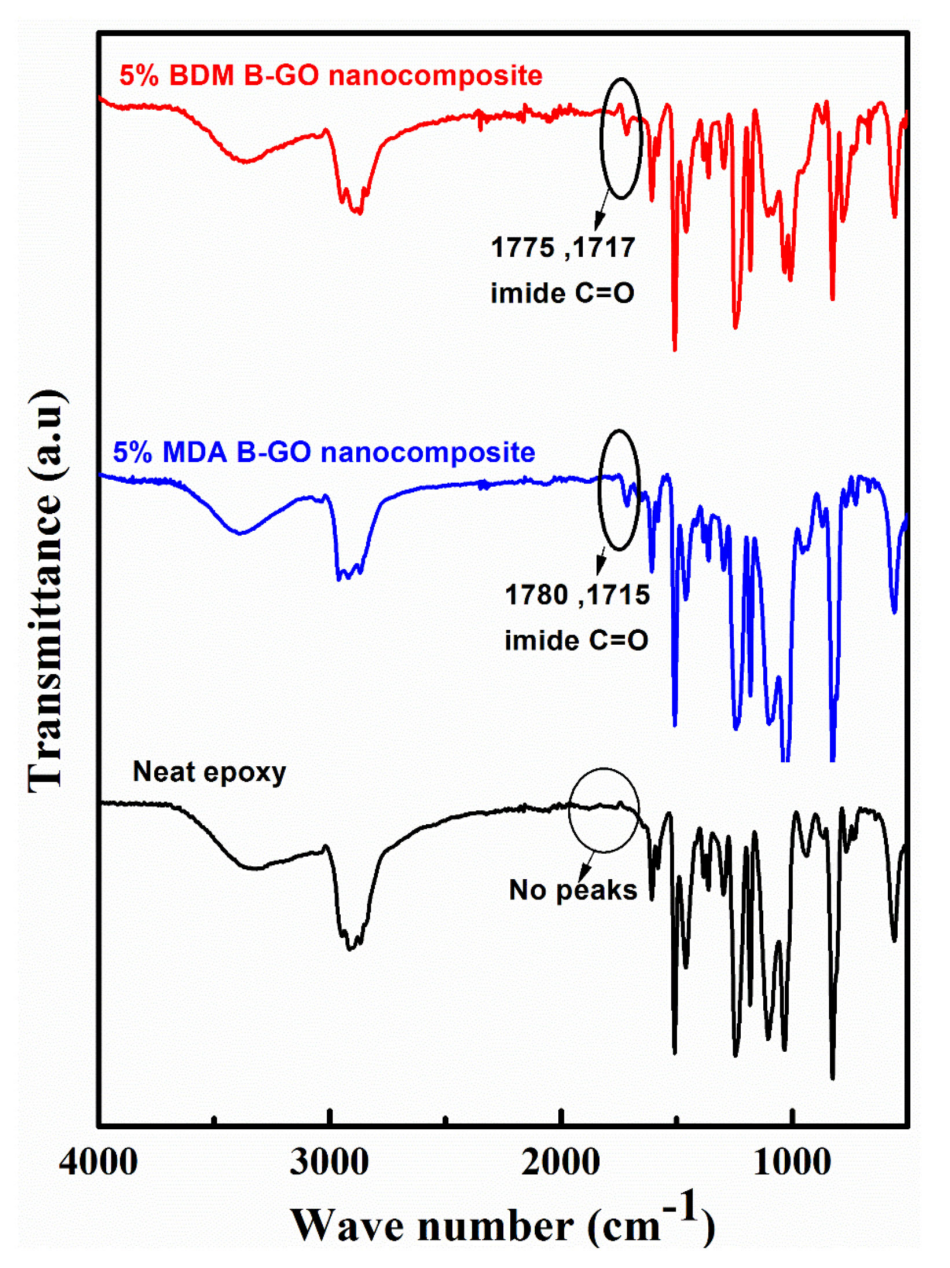
FTIR spectra of neat epoxy and 5% MDA-B-GO/ BDM-B-GO-epoxy nanocomposite.

FTIR spectra of nanocomposites and neat epoxy films are shown in Figure 4. In the FTIR spectra of MDA-B-GO-epoxy and BDM-B-GO-epoxy nanocomposites, characteristic stretching vibration peaks of imide carbonyl were observed, which confirmed the homogenous dispersion of oligoimides in the epoxy matrix. These peaks of the imide carbonyl group appeared at 1780 and 1715 cm^−1^ in the FTIR spectrum of MDA-B-GO, and at 1775 and 1717 cm^−1^ in the case of BD B-GO. However, no such characteristic peak of imide carbonyl group was observed in the FTIR spectra of neat epoxy. Moreover, the characteristic peaks of −NH_2_ group disappeared in the FTIR spectra of nanocomposites. Initially, these peaks were present in the FTIR spectra of MDA-B-GO and BDM-B-GO, as shown in Figure 1. This clearly shows that the terminal −NH_2_ groups of oligoimides, present on the surface of GO, were utilized in the curing of epoxy resin leading to the creation of strong interfacial bonding of fillers with epoxy matrix and ensuring uniform dispersion of GO in the epoxy matrix [17,18].

### 4.2. Morphological Studies

**Figure 5:**
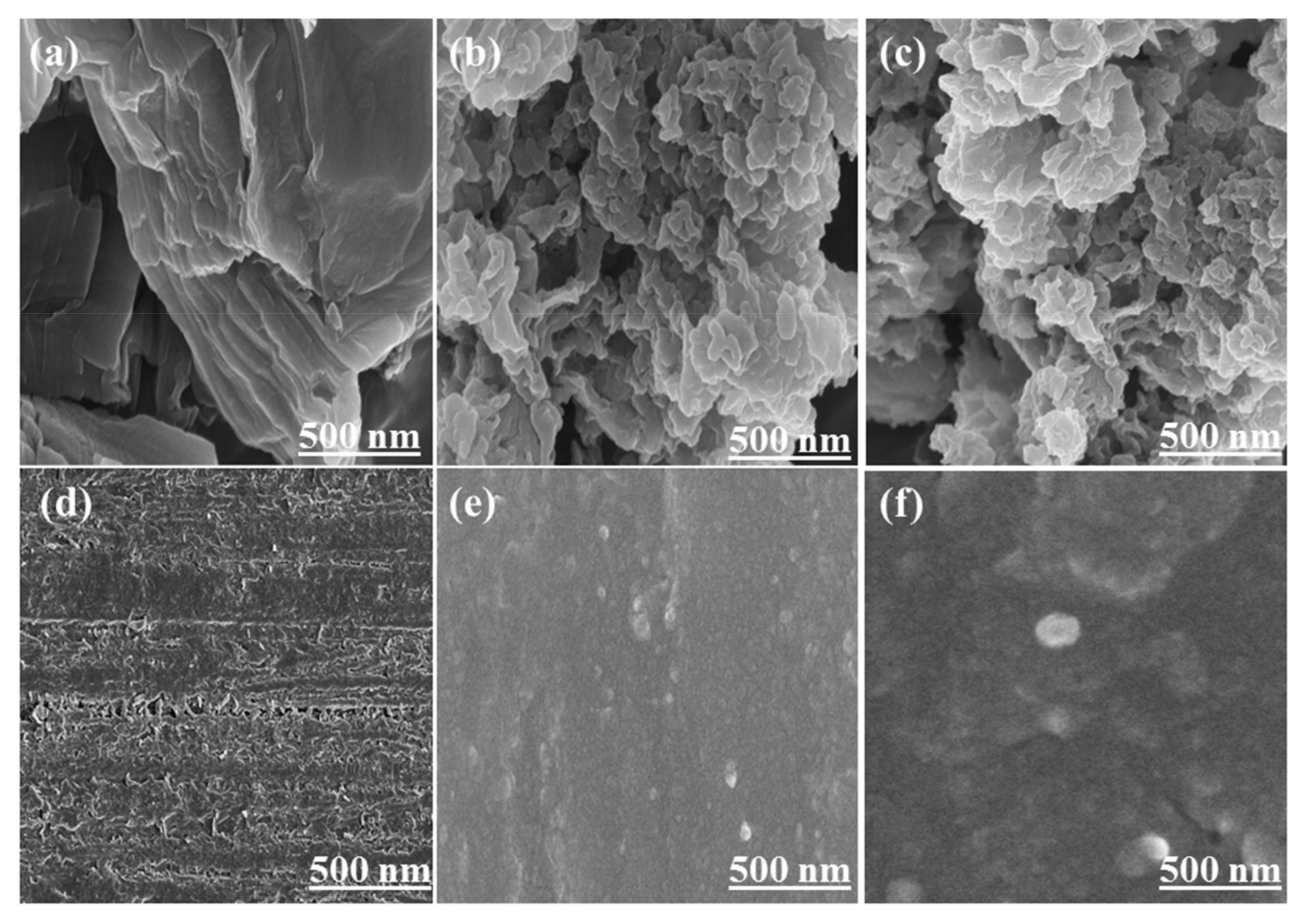
FESEM images of (**a**) pristine GO (**b**) MDA-B-GO (**c**) BDM-B-GO (**d**) neat epoxy. (**e**) 5% MDA-B-GO nanocomposite and (**f**) 5% BDM-B-GO nanocomposite.

The morphologies of pristine GO, both types of modified GOs, neat epoxy, and nanocomposite films were evaluated by FESEM, as shown in Figure 5. Pristine GO appeared as a stacked, sheet-like crystalline form of carbon (Figure 5a). Whereas, GO sheets were exfoliated and acquired sponge-like morphology in the case of MDA-B-GO and BDM-B-GO (Figure 5b,c). This morphological change could be attributed to surface modification of GO. This behavior of GO, due to surface modification, was also reported by Wang et al. [19] and Chandrasekaran et al. [20]. In the case of neat epoxy films, Figure 5d shows that they had flat and characteristically brittle surfaces. 

On the other hand, SEM images (Figure 5e,f) of epoxy-GO nanocomposite films exhibited denser morphology and uniform dispersion of modified GO in the matrices. This could be attributed to enhanced interfacial interaction between the oligoimide (present on the surface of GO) and the epoxy matrix owing to the curing nature of the terminal amino group of oligoimide moieties. Thus, after chemical modification of GO, it was easy to prevent the agglomeration of GO sheets in the epoxy matrix. 

**Figure 6:**
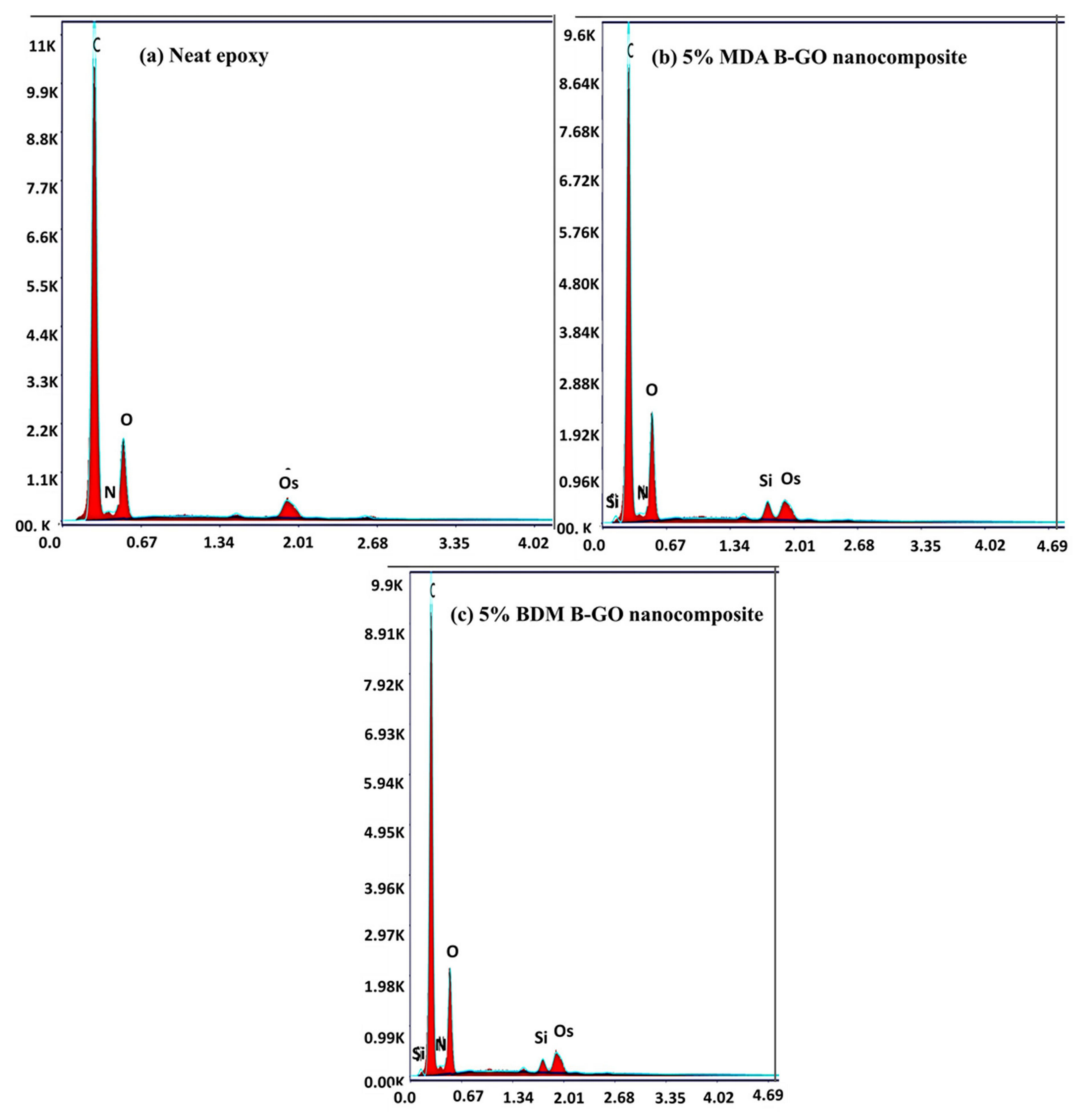
EDX spectra of (**a**) neat epoxy (**b**) 5% MDA-B-GO nanocomposite and (**c**) 5% BDM-B-GO nanocomposite.

Moreover, the EDX spectra of neat epoxy and nanocomposite films provided further confirmation of uniform dispersion of modified GO in the epoxy matrix. The silicon atom, which was only present in the oligoimide moieties, due to modification with APTMS, can be clearly observed in the EDX spectra of epoxy-GO nanocomposites (Figure 6b,c). The silicon atom was absent in the EDX spectrum of neat epoxy, as is obvious from Figure 6a, confirming the successful modification of, and uniform dispersion of, GO in the epoxy matrix. The relative percentages of different elements like carbon, nitrogen, oxygen, and silicon obtained from the EDX analysis of neat epoxy and its nanocomposites are given in Table 4. 

**Figure 7:**
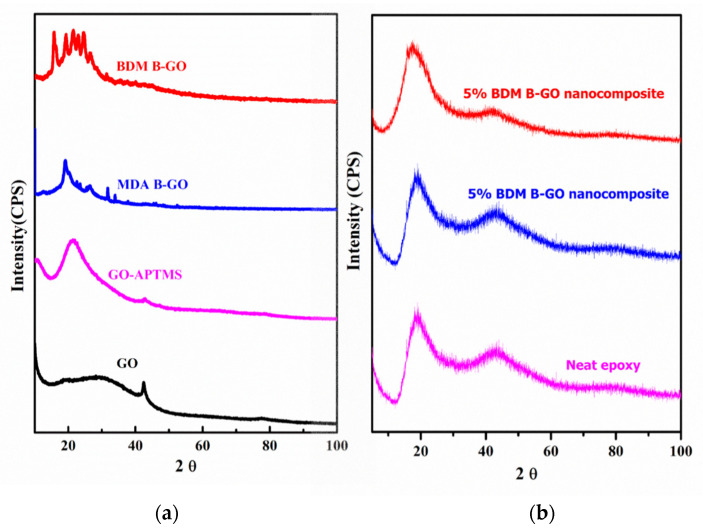
XRD pattern of (**a**) GO, GO-APTMS, MDA-B-GO and BDM-B-GO oligoimides (**b**) Neat epoxy and its nanocomposites.

Notably, for further verification of filler exfoliation and uniform dispersion in epoxy matrix, XRD analysis of GO fillers, the neat epoxy, and nanocomposites was carried out, and the results presented in Figure 7. It can be seen that peak shape and multiplicity were much different in the diffractograms of MDA-B-GO and BDM-B-GO than that of pristine GO, which can be associated with grafting of oligoimides chains on the surface of GO [21]. The diffractogram of neat epoxy showed two broad peaks near 20 and 45° that were attributed to the amorphous nature of the epoxy matrix. It was notable that the diffraction patterns of all the epoxy-GO nanocomposites were similar to that of neat epoxy. It was likely that MDA-B-GO and BDM-B-GO sheets were well intercalated, fully exfoliated and homogenously dispersed in the epoxy matrix. Resultantly, the original diffraction pattern of neat epoxy remained intact [22].

## 5. Thermal Properties

### 5.1. TGA Analysis

**Figure 8:**
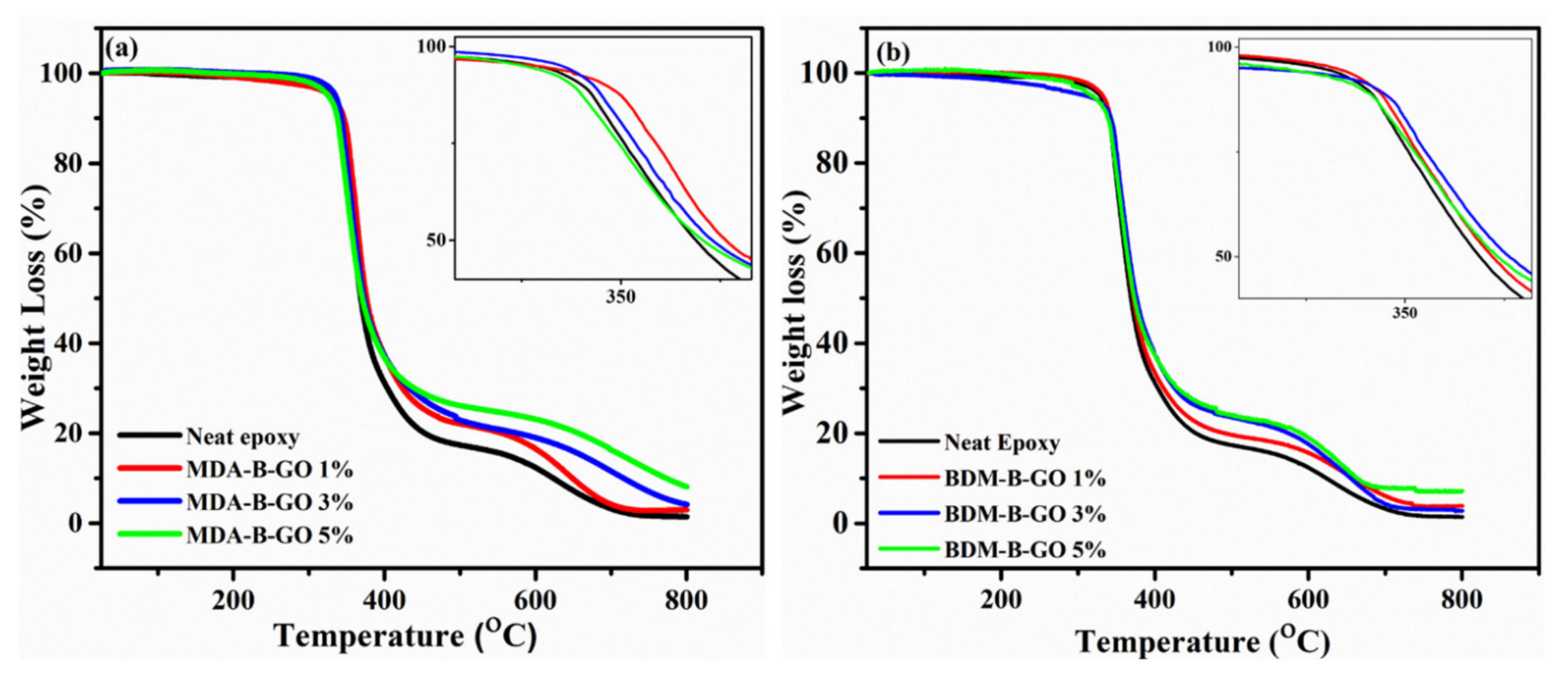
TGA thermograms of (**a**) Neat epoxy and its MDA-B-GO nanocomposites (**b**) BDM-B-GO nanocomposites (**Inset:** Thermal degradation pattern between 300 °C to 380 °C).

Thermal stability of the nanocomposites and the neat epoxy film were analyzed by thermogravimetric analysis to explore the effect of modified GO and its miscibility with the epoxy matrix. TGA thermograms are shown in Figure 8.

TGA analysis was carried out in a nitrogen environment with a heating rate of 10 °C. The TGA thermograms showed a two steps thermal degradation pattern. The first mass loss was observed near 300 °C, which might be related to the degradation of the pendant chain of the polymer matrix, and the second stage of weight loss might be due to the decomposition of the main chain of the epoxy [23]. There were some irregularities in the values of IDT, *T*_10_, and *T*_50_, which could be due to the decomposition of labile oligoimide chains coupled with some residual solvent and moisture in the analyzed samples. However, *T*_max_ values and residual yields gradually increased with increase in filler loading. The thermal decomposition temperature *T*_max_ for neat epoxy was 626 °C, while for the MDA-B-GO-epoxy nanocomposites and BDM-B-GO-epoxy nanocomposites they were 148 °C and 37 °C, respectively, higher in comparison. The higher thermal stability of MDA-B-GO-epoxy nanocomposites than BDM-B-GO-epoxy nanocomposites could be assigned to higher solubility of MDA diamine. GO functionalized with oligoimide groups was well mingled and well adhered to the epoxy matrix due to chemical bonding between both domains, which imparted greater thermal stability. This chemical bonding was achieved by the ring opening reaction of amino groups of oligoimides with epoxy matrix. Similarly, both nanocomposites provided higher residual yields at 800 °C in contrast to neat epoxy. The delayed decomposition process of the nanocomposites illustrated that incorporation of modified GO hindered the oxygen diffusion into the epoxy matrix leading to reduction in degradation at higher temperature. Thus, it could be inferred that MDA-B-GO and BDM-B-GO can act as effective heat and mass transport barriers due to stronger bonding with the epoxy matrix, and, accordingly, are more capable of conserving the matrix.

Previously reported epoxy/modified GO nanocomposites [15] were not as thermally stable as the MDA-B-GO-epoxy nanocomposites and BDM-B-GO-epoxy nanocomposites in terms of IDT, *T*_10_, *T*_50_, *T*_max_, and *R*_800_. Thus, it can be concluded that both MDA-B-GO-epoxy nanocomposites and BDM-B-GO-epoxy nanocomposites have greater potential as thermal conductors, in comparison to their previously reported counterparts [15].

The data derived from the thermogravimetric curves of neat epoxy and its nanocomposites are provided in Table 5 and clearly show enhancement of T_max_ values in both types of the nanocomposites.

### 5.2. DCS Analysis

**Figure 9:**
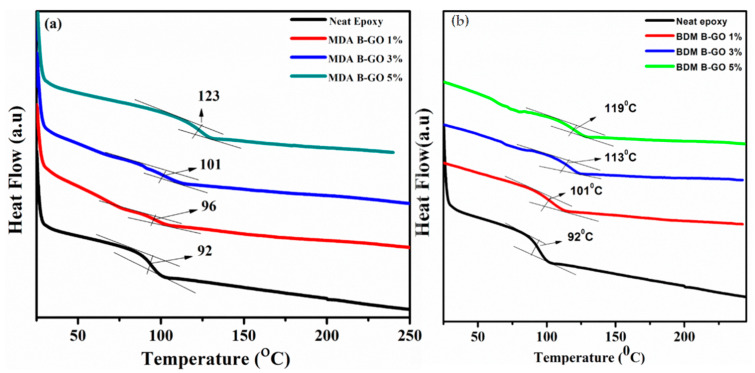
DSC thermograms of (**a**) Neat epoxy and MDA-B-GO-epoxy nanocomposites. (**b**) Neat epoxy and BDM-B-GO-epoxy nanocomposites.

Similarly, DSC analysis was carried out to access the T_g_ and, thereby, to check the thermal performance of the prepared epoxy-GO nanocomposites. The DSC thermograms of the two types of nanocomposites are presented in Figure 9.

These thermograms exhibit that the *T*_g_ values increased with increase of the filler contents. The *T*_g_ value of neat epoxy was found to be 92 °C, while for MDA-B-GO-epoxy nanocomposites and their BDM-GO based counterpart the *T*_g_ values were 123 °C and 119 °C, respectively, at 5 wt % loading of filler. However, at 1 and 3 wt % loading the *T*_g_ values were higher for BDM-GO-epoxy nanocomposites than for MDA-B-GO based nanocomposites, which could be attributed to more polar groups in the structure of BDM diamines than MDA. Thus, oligoimides derived from BDM can have greater inter-chain connectivity than MDA-oligoimides. Consequently, the former imparted greater *T*_g_ to resultant nanocomposites at 1 and 3 wt % filler loading. However, at 5 wt % filler contents, the higher solvent miscibility of MDA diamine than BDM compensated for lower chain interactions in its oligoimide. So, at higher filler loading MDA-B-GO-epoxy nanocomposites had higher *T*_g_ values. This linear increase in the *T*_g_ values of epoxy nanocomposites upon addition of modified GO can be credited to uniform dispersion of GO into the epoxy matrix, which, in turn, was made possible by strong connectivity between filler and matrix.

In various previous reports [12,24,25,26] phase disruption between epoxy matrix and GO filler occurred. This was associated with lower interfacial interactions between the two phases, which hindered the addition of GO over about 1 wt %. In the presented work, we achieved uniform distribution of modified GO into epoxy matrix up to 5 wt % without phase disruption, which was evident from the gradual increase in *T*_g_ values and other thermal properties.

Similarly, MDA-B-GO-epoxy nanocomposites and BDM-B-GO-epoxy nanocomposites can also be compared with previous reports [15] in terms of *T*_g_. Here, MDA-B-GO-epoxy nanocomposites and BDM-B-GO-epoxy nanocomposites exhibited values of Tg as high as 123 °C, whereas in the previous report it was 100 °C. Again, it can be inferred that MDA-B-GO-epoxy nanocomposites and BDM-B-GO-epoxy nanocomposites have superior properties as compared to previously reported epoxy/GO nanocomposites [15].

### 5.3. Thermal Conductivity

**Figure 10:**
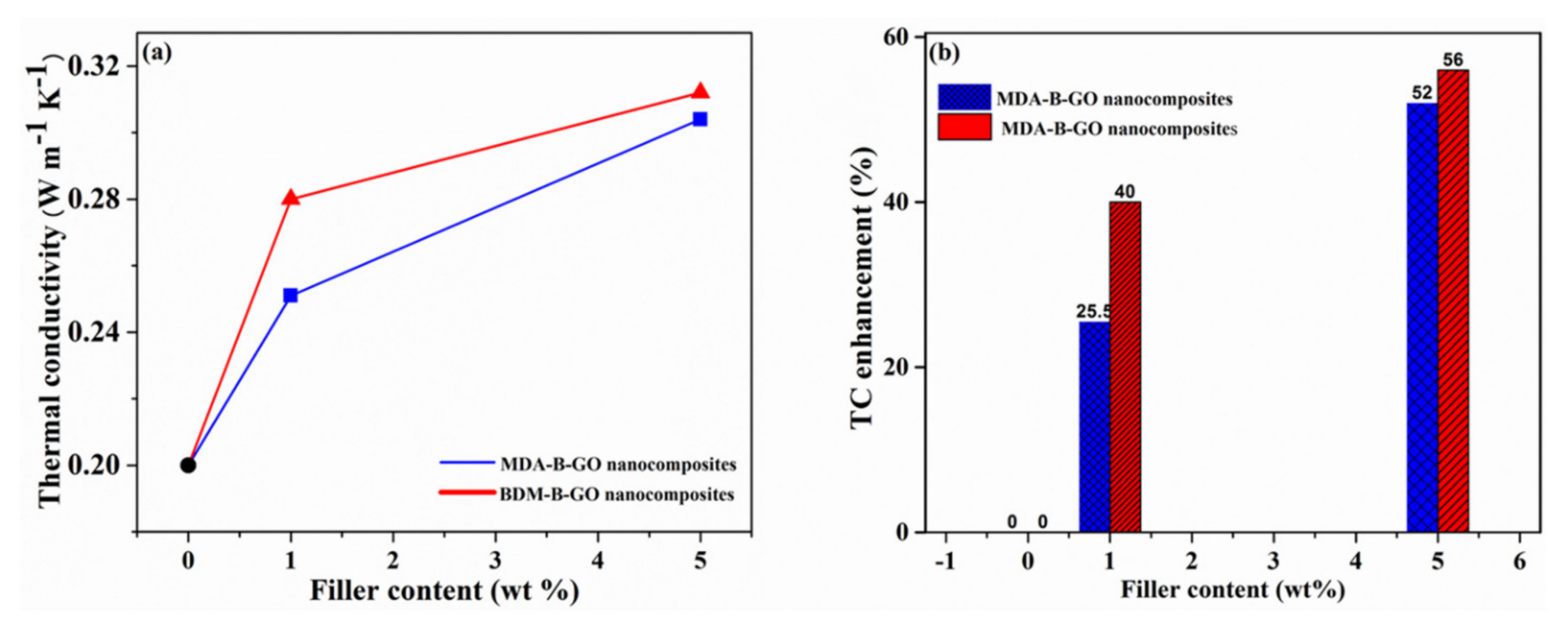
(**a**) Thermal conductivities of nanocomposites (**b**) % Thermal conductivity enhancement in nanocomposites.

Thermal conductivity of the prepared nanocomposites was determined by the hot disc method and the results are presented in Figure 10. Thermal conductivity of neat epoxy was found to be 0.2 W m^−1^ K^−1^, which increased constantly and reached a value of 0.304 W m^−1^ K^−1^ for MDA based nanocomposites and 0.311 W m^−1^ K^−1^ for BDM based nanocomposites, at 5 wt % filler loading in both cases, as shown in Figure 10a. In the former case, thermal conductivity improved by 52%, whereas it was by 55.5% in the latter case, which is depicted in Figure 10b. BDM-B-GO-epoxy nanocomposites possessed higher thermal conductivity, in comparison to MDA-B-GO-epoxy nanocomposites because of their high polar nature and the stiffer backbone of BDM diamine [27,28].

It can be inferred that fully exfoliated GO sheets, which possess excellent thermal conductivity, upon uniform distribution into epoxy matrix developed an interconnecting network, which could efficiently conduct heat [29].

### 5.4. Mechanical Properties

The mechanical properties, like hardness, elastic modulus and plasticity index, of the nanocomposites were determined by nanoindentation using the Oliver and Phar method [30].

**Figure 11:**
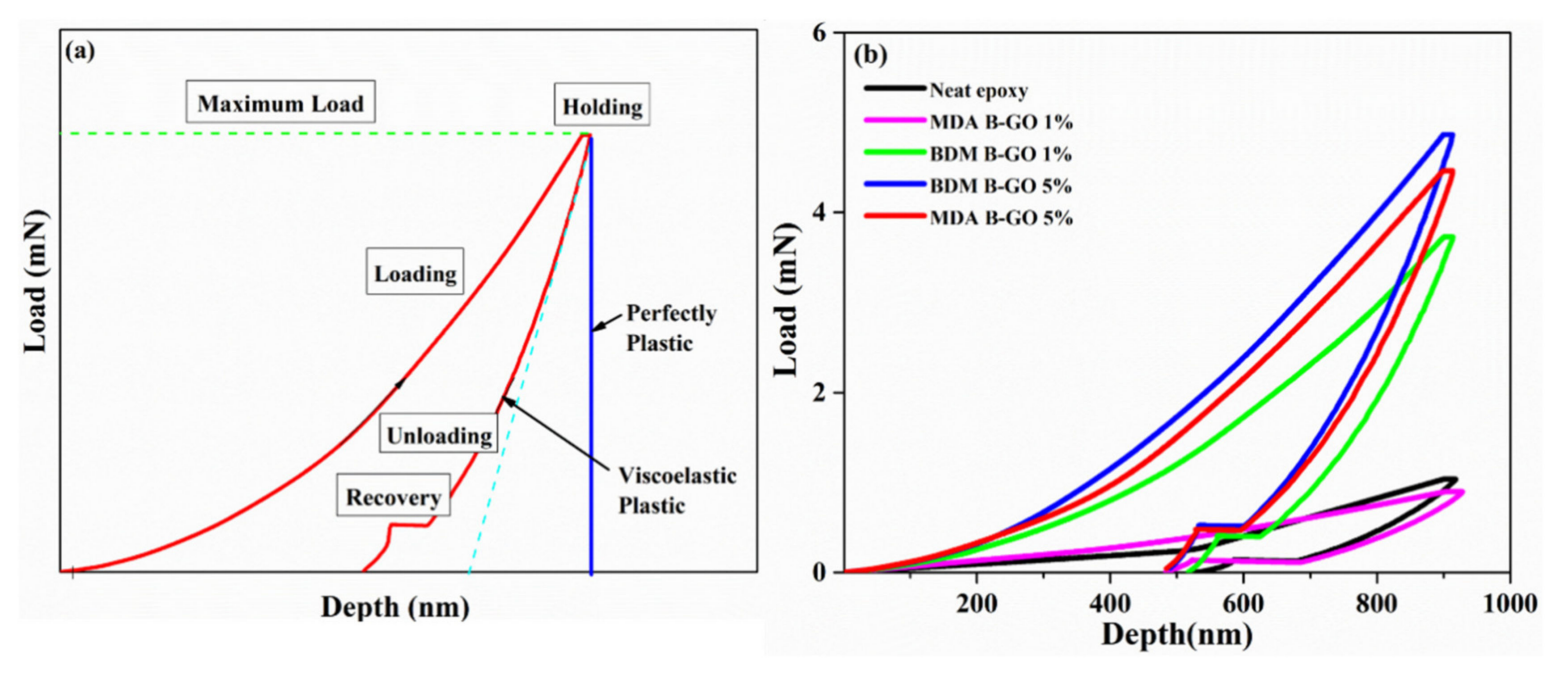
(**a**) Representative nanoindentation curve (**b**) Indentation curves for neat epoxy and its nanocomposites.

**Figure 12:**
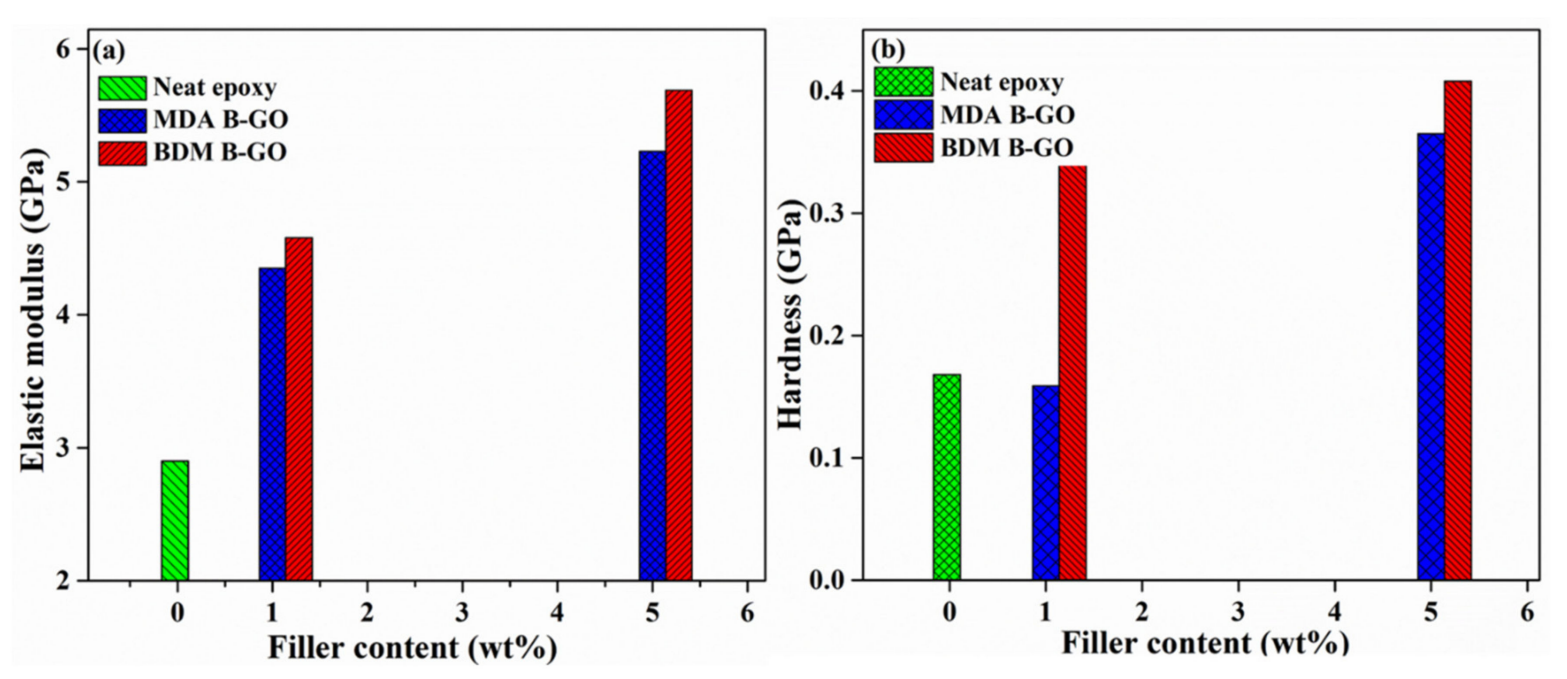
(**a**) Elastic modulus and (**b**) Hardness of neat epoxy and its nanocomposites.

Typical indentation curves of neat epoxy and its nanocomposites are shown in Figure 11, whereas derived data is given in Table 6. The hardness value (H) indicates the plastic behavior of the material and is attained by dividing the value of the applied strain with the projected area [30,31,32,33]. Young’s modulus (E) gives the elastic nature of any material and it is acquired through the slope of the stress-strain curve during the unloading process. The hardness and elastic modulus values for neat epoxy were observed to be 0.168 GPa and 2.90 GPa, respectively. The average of numerical values of elastic modulus and hardness are shown in Table 6 and Figure 12. It can be seen that for 5 wt % MDA-B-GO nanocomposite, the hardness value increased 116% while the value of elastic modulus increased 80% as compared to the neat epoxy. For the 5% BDM-B-GO nanocomposite, a significant increase of 142% of the hardness value and 96% of elastic modulus was observed in contrast to the neat epoxy.

Plasticity index is another parameter that determines relative elastic and plastic behavior of any material [34]. This parameter possesses a range between 0 (completely elastic) to 1 (completely rigid plastic) [35]. The plasticity index of the neat epoxy was found to be 0.60. In the case of 5% MDA-B-GO nanocomposite, the plasticity index was observed to be 0.53, while for 5% BDM-B-GO nanocomposite, its value was 0.52, due to the relatively flexible nature of diamine BDM. It can be concluded from the data of plasticity index that GO, which contained oligoimide chains on its surface, was incorporated successfully into the epoxy matrix leading to a decrease in its plastic behavior and an increase in the elastic nature.

From the data obtained through nanoindentation, it can be observed that the hardness value is significantly enhanced for 5 wt % BDM-B-GO-epoxy nanocomposite.

These results were in a good agreement with the previously reported work of Ribeiro et al. [26] where they prepared epoxy-GO nanocomposites. In their work, GO was modified by the addition of tetraethylenepentamine (TEPA) to its surface. They reported a 143% increase in hardness value for the nanocomposites. In our research work, along with improvement in the thermal conductivity, the elastic modulus of the MDA-B-GO and BDM-B-GO-epoxy nanocomposites also increased by 80% and 96%, respectively.

## 6. Conclusions

In this study, two different types of surface modified GOs, MDA-B-GO and BDM-B-GO, were prepared by modifying oligoimide chains on the GO surface. Conjugating oligoimides was achieved by reacting BTDA with MDA diamine for the MDA-B-GO, and BTDA and BDM diamine for the BDM-B-GO. The successful functionalization of GO, in both cases, was confirmed by FTIR and XPS analysis. The property of modified GO to cure epoxy resin was assessed by DSC analysis. Then, two different series of epoxy nanocomposites were prepared by dispersing MDA-B-GO and BDM-B-GO into the epoxy matrix separately. One series was coded as MDA-B-GO-epoxy nanocomposites and the second series as BDM-B-GO-epoxy nanocomposites. For property evaluation, neat epoxy nanocomposites were also prepared by curing epoxy resin with a commercially available amine hardener Aradur-22962. Thermal conductivity, thermal stability, and mechanical properties of these nanocomposites and the neat epoxy were evaluated by relevant analysis techniques. Thermal analysis showed that parameters for thermal stability, like Tmax, residual yield, and *T*_g_, were increased significantly for both types of nanocomposites at 5 wt % loading of respective GO filler, in comparison to neat epoxy. Importantly, the thermal conductivities of 5% MDA-B-GO-epoxy nanocomposite and 5% BDM-B-GO-epoxy nanocomposite were enhanced by factors of 52% and 56%, respectively, as compared to the neat epoxy. The mechanical properties of nanocomposites and neat epoxy were evaluated in terms of elastic modulus, hardness, and plasticity index. The values for hardness and elastic modulus increased 116% and 80%, respectively, for 5 wt % MDA-B-GO-epoxy nanocomposite, while increases of 142% and 96% were observed in hardness and elastic modulus, respectively, for 5 wt % BDM-B-GO-epoxy nanocomposite, in comparison to neat epoxy. Similarly, both types of nanocomposites exhibited a remarkable decrease in plasticity index, in comparison to neat epoxy. This significant improvement in thermal and mechanical properties of GO-epoxy nanocomposites over neat epoxy were attributed to oligoimide functionalized GO, which, upon uniform dispersion, cured the epoxy rings via pendant amino groups. These nanocomposites showed outstanding high thermal conductivity and mechanical properties, and could be utilized for the development of high-performance epoxy polymers in various applications, including a building block of electrical devices.

## Figures and Tables

**Table 1:** Codes and amounts of fillers in epoxy matrix.

Nanocomposites Type	Codes	Filler %	Amount of Total Mixture (g)	Amount of Epoxy (g)	Amount of Hardener (g)	Amount of Filler (g)
	Neat epoxy	0	3.75	3.0000	0.7500	0
MDA-B-GO-epoxy nanocomposites	MDA-B-GO 1	1	3.75	2.9700	0.7425	0.0375
	MDA-B-GO 3	3	3.75	2.9100	0.7275	0.1125
	MDA-B-GO 5	5	3.75	2.8500	0.7125	0.1875
BDM-B-GO-epoxy nanocomposites	BDM-B-GO 1	1	3.75	2.9700	0.7425	0.0375
	BDM-B-GO 3	3	3.75	2.9100	0.7275	0.1125
	BDM-B-GO 5	5	3.75	2.8500	0.7125	0.1875

**Table 2:** XPS data of GO, GO-APTMS, MDA-B-GO, and BDM-B-GO.

Graphene Type	% C	% O	% N	% Si	C:O	C:Si
GO	67.65	32.35	-	-	2.09	-
GO-APTMS	54.04	23.29	10.18	12.49	2.33	4.32
MDA-B-GO	78.08	14.37	5.74	1.81	5.43	43.13
BDM-B-GO	74.98	16.85	4.01	4.16	4.44	18.02

**Table 3:** Curing data of epoxy with aradur-22962 and oligoimides.

Sample	T_max_ (°C)	∆H (mJ·g^−1^)	% Reduction in ∆H
Epoxy cured with hardener Aradur-22962	106.6	166.79	-
Epoxy cured with 5 % MDA-B-GO	101.2	39.01	76.6
Epoxy cured with 5 % BDM-B-GO	92.5	90.53	45.7

**Table 4:** Percentages of elements in neat epoxy and its nanocomposites.

Element	Neat Epoxy		MDA-B-GO-Epoxy Nanocomposite		BDM-B-GO-Epoxy Nanocomposite	
	Weight %	Atomic %	Weight %	Atomic %	Weight %	Atomic %
C	77.99	82.36	73.17	78.98	74.37	79.88
N	1.72	1.56	1.05	0.98	0.97	0.90
O	20.29	16.08	23.28	18.88	22.77	18.36
Si	-	-	2.53	1.17	1.89	0.87

**Table 5:** TGA data of neat epoxy and MDA-B-GO/ BDM-B-GO-epoxy nanocomposites.

Type of GO-Epoxy Nanocomposites	GO Filler Content (%)	IDT (°C)	T_10_ (°C)	T_50_ (°C)	T_max_ (°C)	R_800_ (%)
	0 (Neat Epoxy)	294	340	368	626	1.34
	1	263	346	377	653	3.0
MDA-B-GO-epoxy nanocomposites	3	316	343	373	720	4.1
	5	300	337	371	774	7.70
	1	308	342	373	657	2.89
BDM-B-GO-epoxy nanocomposites	3	287	343	376	655	3.81
	5	218	338	372	663	7.20

IDT = Initial Degradation Temperature (Temp of 2% weight loss); *T*_10_ = Temperature at which 10% weight loss observed; *T*_50_ = Temperature at which 50% weight loss observed; *T*_max_ = Temperature at which maximum weight loss observed; *R*_800 =_ Residual weight at 800 °C.

**Table 6:** Mechanical properties of neat epoxy and its nanocomposites.

Type of GO-Epoxy Nanocomposites	Filler Content (Wt %)	Hardness (GPa)′	Elastic Modulus (GPa)	Plasticity Index
Neat Epoxy	0	0.168	2.90	0.60
MDA-B-GO-epoxy nanocomposites	1	0.159	4.35	0.57
	5	0.364	5.23	0.53
BDM-B-GO-epoxy nanocomposites	1	0.36	4.58	0.57
	5	0.408	5.69	0.52
